# Testing the feasibility of the Dignity Therapy interview: adaptation for the Danish culture

**DOI:** 10.1186/1472-684X-9-21

**Published:** 2010-09-22

**Authors:** Lise J Houmann, Susan Rydahl-Hansen, Harvey M Chochinov, Linda J Kristjanson, Mogens Groenvold

**Affiliations:** 1 Dept. of Palliative Medicine, Bispebjerg Hospital, Copenhagen, Denmark; 2 Manitoba Palliative Care Research Unit, Dept. of Psychiatry, University of Manitoba, Winnipeg, Canada; 3 WA Centre for Cancer & Palliative Care, Curtin University of Technology, Perth, Australia; 4 Institute of Public Health, Copenhagen, Denmark

## Abstract

**Background:**

**'**Dignity Therapy' (DT) is a brief, flexible intervention, which allows patients to complete an interview and create a document regarding their life, identity and what they want to leave in writing for their loved ones. DT is based on the DT Question Protocol. Developed and tested in English speaking settings, DT has proven to be a feasible and effective way to enhance patient dignity, while diminishing suffering and depression. The aim of this study was to test the acceptability and feasibility of the DT Question Protocol among Danish health professionals and cancer patients, and to obtain preliminary estimates of patient uptake for DT. These results will be used to inform a larger evaluation study.

**Method:**

Ten professionals were interviewed about their perception of DT and the Question Protocol. It was then tested with 20 patients at two palliative care sites and one gynecologic oncology department. Data was analyzed using content analysis techniques to evaluate the protocol for relevance, acceptability and comprehension. The interest and relevance of the intervention was also determined by examining the preliminary participation rate.

**Results:**

Overall, DT was perceived to be comprehensible and relevant. Professionals highlighted six concerns that might warrant modification. These issues were examined using patient data. Some of their concerns overlapped with those raised by the professionals (e.g. *'unacceptable self-praise' *and '*interference with the lives of others'*). Tailoring DT to Danish culture required easily accommodated adjustments to the procedures and the DT Question Protocol. Some concerns expressed by health professionals may have reflected protectiveness toward the patients. While the intervention was relevant and manageable for patients admitted to palliative care, DT was less easily implemented at the gynecologic oncology department.

**Conclusion:**

Based on patients' and professionals' reaction to the DT Question Protocol, and based on the preliminary proportion of participants accepting DT, the DT question protocol - with minor adaptations - appears to be a manageable, acceptable and relevant intervention for Danish patients admitted to palliative care.

## Background

Although palliative care is meant to "*provide... spiritual and psychosocial support from diagnosis to the end of life and bereavement"*, there are few tested, systematic interventions available to address psychosocial and existential sources of distress among cancer patients admitted to palliative care [1]. Interventions targeting end-of-life distress are therefore highly relevant, to help patients live as fully as possible and to support the bereaved.

**Figure 1:**
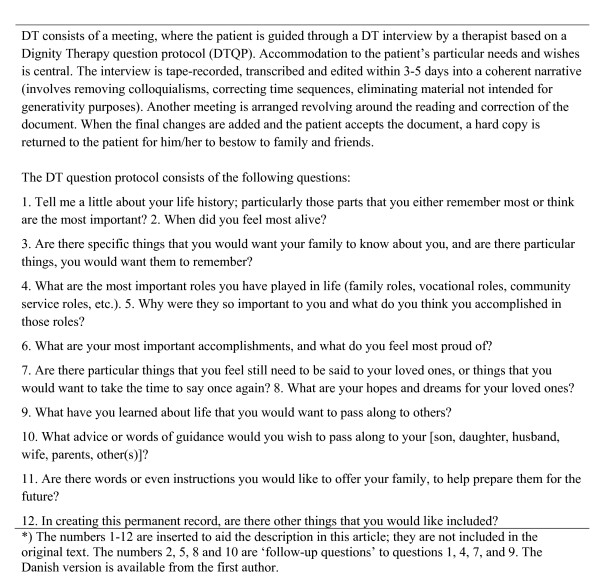
Dignity Therapy and the Dignity Therapy Question Protocol*.

Dignity Therapy (DT) was developed by Chochinov and colleagues based on their previous research on the concept of dignity [2-4]. DT is based on an empirical model of dignity in the terminally ill, which delineates what influences an individual's sense of dignity. The purpose of DT is *"to decrease suffering, enhance quality of life, and bolster a sense of meaning, purpose and dignity" *[5]. Dignity Therapy employs a narrative approach and contains elements similar to Life Review and reminiscence, with its focus on letting the patient find meaning and reconciliation through examining past experiences and achievements, and making amends with or carry out unfinished business [6-9]. It also contains elements from meaning-centered therapies, in terms of creating legacy [10-14]. Further, DT focuses on meaning-making, by inviting patients to reflect on what is important to them. The therapeutic stance of DT is one of unconditional positive regard, as per other supportive therapies [15-17]. The strength of DT lies in the way it combines these elements in a fashion that is clearly described in a manual. Furthermore, DT is specifically tailored to patients living under conditions of severe illness, including heavy symptom burden, psychosocial and existential distress, and physical limitations. Guided by the Dignity Therapy question protocol (DTQP) [5], DT constitutes a distinct and innovative approach (figure 1) that can be conducted at the bedside and completed within days, making it particularly suitable for the palliative care setting. Results from 100 patients, living in Canada and Australia, demonstrated significant reduction of depressed mood, sense of suffering and nearly significant improvement in sense of dignity [5]. Between 81-91% of the patients found DT satisfactory and of help to their relatives, and 67-76% of the patients felt it heightened their sense of purpose, meaning and dignity. Interviews with the relatives' after the patient's death supported these findings. Furthermore, relatives reported great appreciation of the 'generativity document' (an edited transcription of DT), which had helped them during their grief [18].

These positive findings provided the basis for implementing and evaluating DT in Denmark. Despite accurate translation of the DTQP, we anticipated that differences in cultural practices and beliefs might influence the reception of DT by Danish patients and their families. Other differences we anticipated included the Danish organization of health care and the education of Danish health care professionals. Thus, one could not know whether an intervention of this kind would be equally successful and meaningful if uncritically applied in the Danish culture.

It was therefore necessary to test the feasibility of DT in a Danish care setting and explore the extent to which adjustments might be necessary, prior to moving into a more formal and extensive evaluation. The aims of this study were to investigate the following questions: 1. How do health care professionals in a Danish palliative care setting view the DTQP? 2. Do Danish patients find the DTQP relevant, comprehensible and acceptable? 3. What proportion of patients is considered eligible for and accept DT?

Thus, this study focused most specifically on the Dignity Therapy Question protocol and the issue of recruitment, rather than the broader evaluation of how patients experienced DT and its various impacts. The emphasis of our research agenda was guided by the EORTC Quality of Life Group's guidelines [19], stating that before starting to use a newly translated questionnaire, they should be tested amongst small patient cohorts.

## Methods

### Study overview

Feasibility was tested in the following ways:

1. Interviews with professionals about their perception of the DTQP.

2. Implementation of DT with patients.

3. Examining the acceptability of the DT question protocol for patients.

### Participants

For the interviews with health care professionals, ten experts representing different professions and institutions with experience in existential, social and psychological issues pertaining to advanced cancer patients were identified. Data from professionals was viewed as 'hypothetical' because these professionals had never been exposed to DT. As such, their impressions were based on exposure to the DT Question protocol, rather than on first hand experience of how this intervention actually affected patients.

The actual feasibility testing took place with the first 20 patients recruited from two palliative care units (a hospice having in-patients and home-care patient and a department of palliative medicine having in-patients, out-patients and home-care patients) and a department of oncology (a gynecological cancer out-patient clinic). The following eligibility criteria were applied: having a defined incurable cancer (palliative care)/relapse after first-line antineoplastic treatment of advanced cancer (oncology), being at least 18 years of age, being informed about the diagnosis and aware of incurable disease, absence of cognitive impairment, and absence of physical limitations precluding participation.

### Dignity Therapy

DT and the DTQP are described in figure 1.

#### Translation

Following the translation procedure of the EORTC Quality of Life-group [20], two native speakers of Danish translated the DTQP independently from English to Danish. Two native speakers of English translated a preliminary consensus version back into English. When agreement between versions was reached, the Danish DTQP was ready for testing.

#### Therapists

Four psychologists conducted and edited the DT interviews. Professor Chochinov trained these individuals by way of an on-site 3 day workshop and feedback on initial transcripts.

#### Implementation

Recruitment procedures and information materials were developed in close collaboration with the clinical staff of the palliative care units and the gynecologic oncology department. The staff was thoroughly and repeatedly informed about the study and a project nurse maintained contact with the staff, who helped identify suitable participants. The project nurse obtained consent from patients.

### Interviews and analysis

Based on the EORTC Quality of Life Group guidelines [20], three themes (comprehension, acceptability and relevance) were included in the semi-structured interviews with professionals. These professionals were presented with the DTQP and asked what they thought about it, whether any of the questions were more relevant than others, and why so.

Also, with a focus on comprehension, acceptability and relevance, patients were invited to share all their thoughts on the DTQP before, during and after the DT-interview.

All interviews were tape-recorded and transcribed verbatim.

The transcripts were subjected to a systematic content analysis [21]. Professionals' transcripts were analyzed with a focus on comprehension, acceptability and relevance. The frequency with which professionals endorsed various opinions about the intervention was also tracked, to reveal general attitudes held by professionals towards the DTQP. The content of the negative comments was grouped into overarching themes of concern: since none of the professionals had prior experience with DT, their concerns were regarded as hypothetical, in need of empirical testing by patients.

In contrast to the professionals who were interviewed about their hypothetical concerns regarding the DTQP, the reactions of patients were tracked during and after actual DT. To measure its success and applicability, we examined both the content of the responses given by patients (qualitative analysis) and the frequency with which DT-questions were asked and answered in the interviews (quantitative analysis). This was undertaken in order to establish the comprehension, acceptability and relevance of DT. DTQP is a flexible framework, which does not require that all questions are asked, or that questions be strictly confined within the framework. Rather the goal of the DT interview is to obtain sufficient material to prepare a 'generativity' document and that the content be guided by the patients individual choices and needs. The interview typically ended when both patient and therapist agreed that enough had been said to create a substantive document. This variance in the use of the questions allowed for a quantitative analysis, because the therapists and patients' selection and answering of questions enabled detection of patterns. These patterns provide insights about the use of the DTQP by a sample of Danish therapists. The number of times patients were presented with each question, the number of times it was asked per patient and the overall ratio between each question being asked and answered were calculated. This was done in order to determine how relevant or useful both therapists *and *patients perceived each item contained within the DTQP.

To understand the potential uptake for DT, we determined the number of patients who were considered eligible, accepted, and completed DT. This was done for palliative care units and for the gynecologic oncology department, respectively.

## Results

### Participants

#### Professionals

We approached 10 health professionals, all of whom agreed to participate. Nine of these key informants worked in palliative care at either a hospital or hospice and one worked at the gynecologic oncology department. The professionals were comprised of four nurses, one psychologist, three physicians and two chaplains.

#### Patients

Of the 20 patients who took part in the study, 12 were from the department of palliative medicine, six from the hospice, and two from the oncology department (Table 1). Four were outpatients, eight were inpatients, and eight were home-care patients seen at home. Gastrointestinal, breast, or gynecological cancer diagnoses were most frequent. Two thirds of participants were women. Median age was 57 years and median survival was 57 days from DT-interview.

**Table 1:** Patient characteristics Patients (N = 20)

Site	
Department of Palliative Medicine	12
Hospice	6
Department of oncology	2
Sex	
Female	13
Male	7
Age	
Mean	60
Median	57
Range	44-88
Place of service	
Inpatients	8
Outpatients	4
Homecare patients	8
**Survival from admittance (days)**	
Mean	203
Median	108
Range	18-775
**Survival from DT-interview (days)**	
Mean	104
Median	57
Range	3-440
**Primary tumour**	
Head-neck	1
Gastro-intestinal	5
Respiratory	1
Breast	4
Gynaecological	6
Prostate	1
Urogenital	1
Lymphoma (non-Hodgkin's)	1

From September 2005 to January 2006, 210 consecutive patients were admitted to the two palliative care units. Hereof 101 were excluded and 109 were eligible, but out of these patients, 35 deteriorated. Of the 74 remaining patients, 31 accepted the intervention but 6 patients deteriorated before initiation. A total of 25 patients completed DT. Seven of these patients are not part of the analyses, because they participated in DT in a different part of the study (not reported here), which commenced after the feasibility testing period. All in all, 18 patients were included in the analysis of feasibility. The staff experienced positive feed back from patients after their participation and the general impression among staff engaged in enrollment of patients was that DT was feasible for use in the two palliative care units.

The experience in the oncology ward was less successful. Out of 71 consecutive patients, 29 were considered potentially eligible and informed about DT. In contrast to palliative care, where patients were often excluded because they were too ill, patients in the gynecologic oncology department were frequently excluded because the staff doubted that the patients had realized that their disease was incurable. Furthermore, several eligible patients were not informed: the staff never 'found the right time to ask them'. Of the 29 invited patients, 10 never responded and 17 refused. Two patients who refused explained that they felt that the intervention was developed for 'more palliative' settings. One patient said: "*Am I this far out now that it is time to write my life testimony!*" Others pointed to bad timing, no need or no energy. Only two of 29 patients accepted and completed DT. These two patients were also included in the analysis of feasibility.

### Professionals' views

Professionals had both positive and negative reactions to the DTQP - see additional file 1: 'Results from feasibility testing of Dignity Therapy *'. The main message in the positive comments was that the professionals liked the questions and found them relevant and important.

The negative comments were grouped into six concerns: (1) concerns that the protocol prompted existential issues that were too confronting, (2) cognitively challenging issues, (3) culturally unacceptable self-praise, (4) potential overlap between questions, (5) inappropriate words/phrases because of cultural meaning, and (6) interference with the lives of others.

*(1) Too existentially confronting issues: *nine professionals thought that five items were too existentially confronting. The idea that the manuscript will outlive the patient is alluded to in questions 3, 7, 10 and 11. The words *'alive'*, *'still' *and '*future' *(questions 2, 7 and 10) also suggest the patients' impending death, and the word '*permanent'*, emphasizes the irreversibility of the task (question 12).

*(2) Cognitively challenging issues: *eight professionals thought that ten of the questions were potentially too demanding for the patients: questions 1 and 3 were viewed as too open-ended and as "two questions in one", which might confuse the patients. The professionals were concerned that the task of defining something as '*most important"*, whether it is events or accomplishments, could be too difficult for some patients (questions 1, 6).

*(3) Unacceptable self-praise: *the words '*accomplishments' *and '*proud' *request the patients to identify their own successes (questions 5, 6), which was seen as potentially culturally inappropriate. Similarly, it was suggested that the request to pass on life-lessons could strike Danes as reflecting an unacceptable, grandiose sense of self (question 9).

*(4) Overlap: *eight professionals thought that seven questions were too similar and overlapping. Question 3 was described as similar to questions 7 and 10, question 7 as similar to question 8, and question 6 as similar to questions 4 and 5.

*(5) Inappropriate words/phrases: *in seven questions, seven professionals viewed words or phrases as potentially inappropriate. '*Life history*' was considered artificial and intellectual (question 1). '*Roles' *could be associated with acting and inauthentic living (questions 4 and 5). It was suggested that to some people, family life is a setting where you relax and do not have to 'perform'. Thus, although suitable for some family activities, the term '*accomplishments' *caused responses such as *'I do not have to accomplish in my family life'* (questions 5, 6). The words '*would want' *and '*would wish' *(questions 9, 10) were thought of as too complicated, the phrase '*words of guidance' *(question 10) was considered too technical, and '*instructions*' (question 11) too practical. Finally, some thought that '*to prepare for the future' *referring to the bereaved was impossible and inappropriate to expect of anybody (question 11).

*(6) Interference with the lives of others: *One professional felt that '*words of guidance' *and '*instructions' *from the patients could be stressful for the receivers if they felt obliged to follow advice they would have refused under other circumstances (questions 10 and 11)

### Findings in the patient data

Patients, for the most part, answered without hesitation, implying that the questions were readily understood and accepted. Despite the specific issues summarized below, no patients indicated that any questions were incomprehensible, irrelevant or inappropriate.

#### (1) Too existentially confronting?

Very little patient data supports the professionals' concern regarding existentially confronting questions. No patients seemed adversely affected or refused to answer question 2; however, the question was only posed to four patients, suggesting that the therapists may have been uncomfortable with the question.

#### (2) Cognitively challenging?

The interviews confirmed that some patients perceived the interview as demanding. In question 1, five patients found it difficult to choose what important life experiences to focus on: ('*I don't know where to begin*', *'Have I remembered it all?')*. However, with encouragement and prompting as outlined in the DT manual, they managed to find a relevant answer except for one patient, who answered the subsequent questions instead. Several patients also expressed concern as to whether they had forgotten to mention anyone, had forgotten important messages, or had formulated messages in a hurtful or offending way (*'Am I doing it right?'*). As per the DT manual, the therapists sought to address these concerns during the interview or in the editing process.

#### (3) Unacceptable self-praise?

As expected by the professionals, given Danish sensibilities to this issue, several patients were reluctant to speak of themselves in positive terms. Two patients refused to describe themselves in question 3, because it was up to the relatives to choose what to remember. One patient said she had not had any roles that she considered to be '*important' *(questions 4 & 5). The term '*accomplished' *in question 5 was systematically skipped by the therapists and when they used it in question 6, two patients were uncomfortable describing anything in their lives in terms of *'accomplishments'*. To soften the wording, the therapists sometimes combined '*proud of' *(question 6) with alternative formulations such as *'...or happy with'*, or reminded the patient what he/she might be proud of. Still, three patients found it difficult to identify with the feeling of pride. Eight patients acknowledged the sense of pride in relation to their children only.

#### (4) Overlap?

One patient expressed a concern with repeating herself and there were no indications of similarity between questions in the other DT interviews. We did not further probe why the one patient was bothered by the repetition of questions, as that would have meant stepping outside of a Dignity Therapy agenda and having them enter into a critique of the protocol itself.

#### (5) Inappropriate words/phrases?

The patients found a few of the translated phrases or words inappropriate. One patient said that '*most alive*' (question 2) led him to talk of his youth, which was not a particularly relevant period to include. Two patients objected to the word '*role' *(question 4 and 5).

#### (6) Interference with the lives of others?

Two patients reacted to the invitation to offer '*words of guidance*,' by saying it was inappropriate to tell others what to do (question 10). For the same reason, five patients reacted against '*instructions'*, indicating that it would be a violation of the free will of the receivers to include instructions in the document. The transcripts showed that the patients often interrupted the therapist while he/she was asking the question, and objected to the suggestion of *instructing *their loved ones. Therefore, the last part of the question encouraging the patients to formulate messages that would be of comfort to the relatives was often not heard by the patients (question 11).

### Other findings

Five patients had difficulties relating to the title, "*dignity therapy" *(particularly the term '*dignity'*). One patient said '*I have never strived for dignity*', another patient said; "*For me the name is wrong. This is my life addressed to my children.' *Three patients said that they could not relate to or understand the term 'dignity', still one of them indicated that the intervention had made her feel more valuable.

Two practical problems occurred. One patient died the day after the DT-interview, and was therefore unable to approve the final document. Still, her relatives adamantly wished to receive the document. After consultation with the local Ethics committee, the document was completed, but potentially controversial elements were removed. Another problem concerned the lack of a designated recipient. A patient lived alone with his mother, but could not think of anyone for whom he wanted to make a document, not even his mother. Although the patient enjoyed the visits from the therapist, the lack of a recipient raised questions about the editing process and the appropriateness of the exercise.

### Quantitative analysis of the DT interviews

The mean number of DTQP questions asked per interview was 6.5 (range 3-11). The three right collums of the table in the additional file 1: 'Results from feasibility testing of Dignity Therapy *' shows the number of patients presented with each question, the mean number of times each question was asked and repeated, and the overall likelihood of a question being answered when asked. While this data was collected with the intention of demonstrating how receptive patients might be to each DT question, the varying degree to which questions were posed also reflects some ambivalence on the part of the therapists to broach these issues. As such, this data needs to be considered within the context of those limitations.

## Discussion

In contrast to the publications describing and evaluating DT in Canada and Australia [5], this feasibility study tested DT in a considerably different culture. Overall, the relevance, comprehensibility, acceptability, and feasibility of DT with Danish patients were demonstrated. However, the study revealed the need for minor adjustments of DT, before larger studies or clinical applications in Denmark could be considered. While some of the changes may be relevant only for Danish patients, others may be of general relevance for clinicians and investigators considering cultural adaptation of Dignity Therapy within their particular locale.

### Recommendations and adjustments to the DTQP

Each of the six areas of concern raised by the professionals and/or patients is important to discuss when considering culturally directed protocol adjustments. Our recommendations are based on the data from this study, and are synchronous with the overall intentions of Dignity Therapy. These recommendations have been developed and vetted by our research group, in close cooperation with all participating therapists.

#### (1) 'Too existentially confronting issues'

It is remarkable that the concerns of DT being too existentially confronting were not confirmed by the patients. This may indicate that the therapists have been successful in adapting the interview to each patient, and confrontation has thus been avoided. Maximal attention must be paid to ensure that the patients are not distressed by the intervention. Therapist must learn how to gently introduce topics that might be emotionally evocative, while always being respectful of the patient's healthy defenses. While a skilled therapist will guide the patient to consider each aspect of the DTPQ, he or she will do so in a fashion that gives the patient complete latitude to shape the interview in ways that are personally meaningful, fulfilling and comfortable.

*Recommendation: *Good DT, like good communication, is always sensitive to individual patient needs. The DTQP is meant as framework and special attention must be paid to adjust the language and content to the patients' level of acceptance. Questions 3 and 7-12 all refer to a future beyond the death of the patient; however, this is by implication, as the words death, dying, terminal or palliative are never used. Therefore, if the patient does not talk openly about death, these questions can instead be worded in terms of a 'here and now' vocabulary (e.g. tell me about some of the important things in your life [rather than focusing on 'remembering']; can we talk about some of the things life has taught you [rather than focusing on lessons to be passed along]). In this way the interview is framed as an *opportunity *to have things written down.

*Adjustments: *Because the meaning of the Danish translation of the word '*alive' *in question 2 was ambiguous and overly confronting, the tense of the verb was adjusted to mean 'vigorous'(as intended in the English version) instead of 'alive as opposed to dead'. '*Still*' was removed in question 7 to reduce the implication of impending death. '*Permanent' *was removed from question 12.

#### (2) 'Cognitively challenging issues'

The patient data confirmed that specific questions may be challenging, although in most instances, not overwhelming. However, this may equally well be a reflection of the perceived importance of the task, the goals which the process may evoke with patients, and, more generally, the difficulty of conveying important memories and messages. These issues highlight the therapists' important role as a facilitator and their ability to be responsive to the patient's energy, concentration abilities and pacing of the interview.

*Recommendation: *It is important to reassure the patient that the DT questions are only a framework, that the creation of a DT document is a task with many solutions, and that the interview is a first step that will be followed by a process of editing. Patients who feel they are being asked to reach too high may be reminded that any reach whatsoever is a success. Superlatives such as 'most important memories' should be de-emphasized and it may be explained that even ordinary memories can be extraordinary, if they are authentic, heartfelt and unique to that individual. If patients worry about omitting important memories, messages, or people, they may be reminded that they can always add this content during the editing process. In case of these worries, the interview can focus on clarifying names, dates and places, before returning to larger content issues.

Finally, patients may be reminded that we can help give the material shape through the process of editing and that they will have a chance to participate in this process by noting things that they would want changed.

*Adjustments: *The term *'feel'*, which in Danish may imply a deeply felt need for disclosure, was changed to '*think*' (question 7). In consideration of those who feared hurting others, the focus on life lessons was highlighted, with less emphasis being given to what they *"would want to pass along to others" *(question 9).

#### (3) Unacceptable self-praise

The findings here strongly suggest that Danish patients are reticent to talk about things that they feel may be perceived as boastful or simply self-praise. Many patients refused using terms such as accomplishments, importance and pride about themselves or their roles in life. This appears to be a clear cross-cultural difference from the Canadian/Australian setting where DT was developed. These Danish experiences may be influenced by the 10 commandments also referred to as the "Jante Law" [22]: 'a pattern of group behaviour towards individuals within Scandinavian communities, which negatively portrays and criticizes success and achievement as unworthy and inappropriate' [23].

*Recommendation: *Based on these experiences, the therapist should always ensure that the patient is made comfortable speaking about himself or herself. This must be done in ways that are culturally acceptable and in accord with the patient's outlook. This can be achieved with a down-to-earth approach, the therapist's reassurance of interest in the patient and a therapeutic stance of positive regard. Patients' attention can also be drawn to aspects of their life story, which deserve to be thought of as significant and worthy of knowing, from the vantage point of the therapist and the patient's family. If patients give negative responses to the word accomplish (question 5), it could be changed into *'what do you think you were able to do OR got done*'.

*Adjustments: *To make question 6 appear less self promoting, '*accomplished' *was changed into '*done' *(yet left unchanged in question 5 due to insufficient data), and '*most proud of' *was changed into *'most happy with'*.

#### (4) 'Overlap'

Several professionals saw the overlap between the questions as problematic, but this did not appear to be problematic for patients. Several things may explain this discrepancy. First, whereas professionals reviewed all questions, patients were only asked selected questions as deemed appropriate from within the DT protocol framework. Second, due to repeated words or phrases, the questions may appear more similar than they actually are. Finally, patients might appreciate the chance to build on their responses, based on questions that are thematically linked.

*Adjustments: *None.

#### (5) 'Inappropriate words/phrases'

Professionals, and to a lesser extent patients, noted a number of instances of inappropriate wording in the Danish translation of the DTQP.

*Recommendation*: Despite the modifications listed below, some patients may still need rewording or explanation for comprehension of specific questions. Dignity Therapy should always be offered in a fashion that makes it accessible and comfortable, irrespective of the cultural context or language in which it is being conducted.

*Adjustments: *The terms '*specific'*, '*particular' *and '*would want' *(questions 3, 7, and 9) were removed from the Danish version to make these questions less formal and less complex. To deemphasize the term '*roles' *(question 4) - which is an uncommon Danish term - and to create more awareness of the examples, the word 'roles' and the brackets were removed from the examples '*e.g. in the family, job wise or in the community etc'*. To shorten question 7, the formulation '*take the time to' *was removed. "*Other things*" in question 12 became "*more*" in the Danish version, which is considered to be more inclusive.

#### (6) Interference with the lives of others

Both professionals and patients reacted to particular words in question 10 and 11 that were considered to be too interfering or demanding on the relatives. This was not the original intent of the questions, which were designed to give patients an opportunity to provide their family members messages of comfort and support.

*Adjustments: *To make the issue of passing on comforting and helpful messages more clear, the first part of question 11 including the word '*instructions*' was changed into *"Is there anything you could say."*

Questions 1, 5, 8, and 10 were not changed in the Danish version as there was no support of the professionals' concerns in the patient data and as we wanted to make adaptations to the DTQP only when necessary.

### Adaptation of DT in general

Even though several Danish patients questioned the term '*dignity*', it is important to note that the term *'dignity*' is not referenced in the DTQP. While it was beyond the scope of this study to address this issue adequately, the patients response suggests that a future study of Dignity Therapy would demand that careful attention be paid to how DT is introduced, ensuring that the language used and the rationale provided not be overly existentially confrontative. In practice, the title would also have to be deemphasized when presenting the intervention, and more emphasis be placed on the content of the intervention.

The strategy implemented to safeguard against disappointed relatives when the patient is unable to complete DT because of deteriorating health, was to simply ask the patient after the interview: "*If you are too ill or unable to complete this document, what would you prefer happened?*" In this way, the patient can decide if the interview should be passed on to family members. This also provides permission for the therapist to edit possible hurtful material, so only appropriate and constructive passages are included. The experience with the patient who had no one to bequeath the document to, highlights the importance of clarifying the recipients of the document with the patient, before commencing the intervention. This avoids hurting those who do not have anyone to give the document to and offering patients alternatives that are personally viable and meaningful.

### Quantitative analysis of the DT interviews

The therapists and patients' selection of questions enabled detection of certain interview patterns.

Therapists frequently asked the questions 1, 4 and 8, whereas there seemed to be a hesitation towards question 2, 5 and 11. Thus question 5 was asked using an alternative wording every time, never in its full length. The same holds for question 11 (asked 8 times, 5 times with alternative wording). While questions 2 and 5 were answered every time, question 11 was answered only 63% of the times asked and sometimes caused some patient discomfort. This again underscores the importance of adapting questions and the language used to pose questions in a fashion that is not overly jarring or existentially confronting. The rather infrequent use of question 2 (asked 4 times) may simply reflect that this is a follow-up question that is rendered superfluous if a full response has already been given.

Patients answered questions 1 and 8 very frequently when asked, whereas other questions were answered only about half of the times they were asked. Thus, the low rate of answering questions 4, 6 and 7 (each dealing with a facet of pride or accomplishments) corresponds with the qualitative analysis that illuminated some patients' objections to aspects of these questions. When asked question 12, patients typically said that they had no more to say. The interview had in most cases covered the most essential topics with the previous questions.

### Feasibility testing of DT in different groups of cancer patients

While the results of this study indicate that DT is feasible in palliative care institutions, the figure of 25 participants out of 74 truly eligible patients also shows that this is not an intervention that is applicable to all patients. Furthermore, a large proportion of the patients is too ill in this period of their illness, and never passes the entry criteria. However, in comparison to the results from the gynecologic oncology department, the discrepancy between how well DT was received by patients was large. This eventually made us cease recruitment at the oncological ward, concluding that this study was unable to establish the feasibility of DT in the non-palliative setting. It should be emphasized that we made an effort to adapt the intervention to this setting (i.e. not referring to incurable disease or death in the presentation, but rather motivating participation with reference to how patients in their situation often reflect about their lives and are occupied with wishes to write down memories). However, this did not have the effect we hoped for among staff, who seemed to become gradually more reluctant in including and informing patients. Thus, even though the prognosis of the referred patients was not much better than that of patients admitted to palliative care, DT did not appear as acceptable in its present research design in this particular oncological setting. These experiences further suggested that a future study of Dignity Therapy will demand that careful attention be paid to how DT is introduced, ensuring that the language used and the rational provided not be overly existentially confrontative.

### Strengths and Limitations of the study

This study did not deal with the feasibility of Dignity Therapy overall, but rather, focused on the elements of the DT interview. Further evaluation of the intervention, including testing the feasibility of the editing process, is needed. However, a major strength of this study is that the feasibility of the DTQP was examined from several angles. The study included examining a professional 'hypothetical perspective' and an 'in-vivo patient perspective', and investigated how the rationale of the DT-interview was perceived in different clinical settings. Together, these data give diverse insights into the reception of DT in a Danish culture. Relatives' views on DT and the DTQP have not been explored in this study, but are important.

It must be kept in mind that professionals usually complimented the overall gestalt of the question, followed by various concerns or specific critique raised afterwards. In the analysis, we focused primarily on the latter, but it should be emphasized that their overall evaluation was highly positive. The answers provided by professionals should be viewed with caution, because they were not directly involved in or acquainted with DT. That said, the concerns raised by professionals helped us structure the analysis of patient data and could be tested, while at the same time, we remained open to issues raised by patients that had not been addressed by professionals.

It should be noted that the strategy of inviting patients to share their thoughts about relevance, comprehension and acceptability led to feedback that was mainly problem focused and often lacking positive comments. When patients found the questions appropriate, they simply proceeded to answer the questions (rather than offering an evaluation). Had we tested the questions independently of carrying out DT, the number of positive responses may have been higher. We decided not to proceed in that way, given that patients had extremely limited time and energy, and testing questions might have taken time away from DT.

In the analysis, it was difficult to determine whether some of the concerns - such as the risk of excessive existential confrontation - were based on a protective or paternalistic stance, rather than being attributed to linguistic or cultural translation issues. Among professionals and staff, there was a general fear of confronting the patients excessively. This suggests that people hold misperception that DT focuses prominently on issues pertaining to death and dying. In order to introduce DT across various settings, the protocol will need to be explained well, and the staff educated that in the hands of a sensitive clinician, death awareness need not be confronted by way of dignity therapy. Clearly, professional education and positive experiences with DT, illustrating its applicability and success with this particular patient population, is required. Without appropriate understanding and buy in on the part of healthcare providers, Dignity Therapy--like any novel therapeutic approach--will not be given its fair chance to mitigate suffering for patients facing life threatening and life limiting conditions. Although very few patients conveyed feeling overly confronted, these issues still need to be broached in future research.

The first author had a dual role as both a researcher and therapist. To mitigate any risk of bias, another researcher (SRH) took part in the qualitative analysis. All authors were involved in formulating the final conclusions and took part in the final write-up. To further minimize bias, the opinions of dignity therapists regarding the DTQP were not included in the professional data. Therapist-to-therapist variation can influence a feasibility study such as this. Four psychologists participated as dignity therapists in this study. Recognizing the important role of the therapists highlights the need to evaluate inter-therapist variation, whether launched in a new country, or when new therapists from different professional backgrounds and institutions within the same country are involved.

The experiences of testing DT with cancer patients in active treatment were limited, making it difficult to draw final conclusions about the feasibility of DT in non-palliative settings. Attention to the recruitment difficulties we encountered and future tailoring of DT to this particular population is warranted.

## Conclusions

This feasibility study, which is based on findings from interviews with professionals, from interview data of patients engaged in DT, and general experiences with implementing DT in different clinical settings, overall demonstrated that Danes admitted to palliative care found DT acceptable, relevant and manageable. However, data also showed that Danes sometimes have resistance towards talking about pride and accomplishments, addressing themselves in outright self-praising terms, as well as reticence imposing their will on the receivers. These findings probably reflect cultural characteristics of Danes, and have led to small revisions to the Danish DTQP. The slightly revised question protocol is now targeted to Danish patients, with the intent and overall form of the original intact. Based on these experiences, it is recommended that Dignity Therapists need to be culturally sensitive when applying this intervention. Based on the conclusion that it is feasible to administer the DTQP for Danish patients admitted to palliative care, we are now proceeding to undertake a formal evaluation of DT in a prospective, longitudinal intervention study.

## Abbreviations

'DT': Dignity Therapy; 'DTQP': Dignity Therapy Question Protocol; 'EORTC': European Organisation for Research and Treatment of Cancer.

## Competing interests

The authors declare that they have no competing interests.

## Authors' contributions

LJH participated in the design of the study, conducted the interviews with professionals, conducted 8 DT 's, analyzed the data and drafted the manuscript. LJK, HMC, SRH and MG participated in the design of the study, supervised the interviews and participated in the analysis and discussion of results and writing of the manuscript. All authors read and approved the final manuscript.

## Pre-publication history

The pre-publication history for this paper can be accessed here:

http://www.biomedcentral.com/1472-684X/9/21/prepub

## Supplementary Material

Additional file 1 **Results from feasibility testing of Dignity Therapy.doc'**. The additional file 1 contains a table depicting the results from the feasibility testing of the DT interviews. It consists of qualitative comments from professionals and patient data from the Dignity Therapy interviews. Furthermore, it contains an overview of the frequency with which questions from the Dignity Therapy question protocol were asked and answered. Click here for file
