# Supplementary material for: Testing the feasibility of the Dignity Therapy interview: adaptation for the Danish culture
Source: BMC Palliat Care. 2010 Sep 22;9:21. doi: 10.1186/1472-684X-9-21 (PMC2954968; doi:10.1186/1472-684X-9-21)
Supplement: Additional file 1 — Results from feasibility testing of Dignity Therapy.doc'. The additional file 1 contains a table depicting the results from the feasibility testing of the DT interviews. It consists of qualitative comments from professionals and patient data from the Dignity Therapy interviews. Furthermore, it contains an overview of the frequency with which questions from the Dignity Therapy question protocol were asked and answered. [file 1472-684X-9-21-S1.DOC]

#### Additional file 1: Results from feasibility testing of Dignity Therapy*

| **Qualitative analysis** |  | **Quantitative analysis** | | |
| --- | --- | --- | --- | --- |
|  |  | Patients asked | Mean per patient  ** | Ratio of answers/ questions  *** |
|  | | | | |
| ***Question 1: Tell me a little about your life history; particularly those parts***  ***that you either remember most or think are the most important?*** | | | | |
| **Considerations:**  (+) Six professionals commented the wording, relevancy and (hypothetic) effect positively.  (-) Five professionals found it challenging by being broad, open-ended and consisting of two parts. They stated that it could be hard to tell what has been '*most'* important in life.  (-) Four professionals found *'life history'* 'artificial and intellectual'. | |  |  |  |
| **Findings:**  (-) Four patients found it difficult to focus on what had been *most* important.  (-)‘Life history’ was exchanged with ‘life’ seven times; content of answers were similar. | | 20 | 1.7 | 88%  (30/34) |
|  | | | | |
| ***Question 2: When did you feel most alive?*** | | | | |
| **Considerations:**  (+) Three professionals found it relevant as a good follow up to question 1.  (-) Three professionals found it confronting due to the past tense and the word 'alive'. | |  |  |  |
| **Findings:**  (-) One patient said he associated ‘most alive’ with his energetic youth, but this was not an important period of his life. | | 4 | 1.0 | 100%  (4/4) |
|  | | | | |
|  | | | | |
| ***Question 3: Are there specific things that you would want your family to know about you,***  ***and are there particular things, you would want them to remember?*** | | | | |
| **Considerations:**  (+) Six professionals underlined the relevancy in terms of the questions’ potential to let the patient focus his answer and direct it at the family.  (-) Four professionals found it confronting by implying the manuscript’s lasting nature beyond the patient’s own existence.  (-) Two professionals found it too open-ended, a two-in-one-question, and too similar to questions 7-11. | |  |  |  |
| **Findings:**  (-) One patient said that she had already talked to her family about it.  (-) One patient objected because what his children would remember him for, was in their own minds. | | 11 | 1.9 | 76%  (16/21) |
|  | | | | |
| ***Question 4: What are the most important roles you have played in life (family roles,***  ***vocational roles, community service roles, etc.).*** | | | | |
| **Considerations:**  (+) Three professionals found it was relevant to frame the patient and his/her life through ‘roles’ and particularly the examples associated with them. Four additional professionals had positive comments to the question.  (-) Five professionals criticized the term ‘*role*’ of being unauthentic, associated with acting, and atypical for a normal everyday Danish setting. Three professionals tried to present alternative formulations without success. | |  |  |  |
| **Findings:**  (-) One patient did not see herself in 'any particular role' and did not ‘feel that way’.  (-) One patient said she had no ‘important’ roles and could not answer. | | 13 | 1.8 | 61%  (14/23) |
|  | | | | |
| ***Question 5: Why were they so important to you and what do you think you accomplished in those roles?*** | | | | |
| **Considerations:**  (+) Four professionals commended question five for being relevant and natural as a follow up to the previous question.  (-) Four professionals found that ‘*accomplishments*’ may be inconsistent with family roles and soft values. It can offend people who think they lack achievements, and it may be perceived as unacceptable to disclose such answers. | |  |  |  |
| **Findings:**  (-) No comments from patients.  (-)Therapists always left out the part "*what do you think you accomplished in those roles*". | | 6 | 1.7 | 100%  (10/10) |
|  | | | | |
| ***Question 6: What are your most important accomplishments, and what do you feel most proud of?*** | | | | |
| **Considerations:**  (+) Six professionals considered it to be a relevant and important question for some patients.  (-) One professional thought that the adjective *'most important'* could overwhelm patients.  (-) Regarding *'accomplishments*', see question 5.  (-) Two professionals said that it might seem wrong to say out loud what you are proud of.  (-) Two professionals found an overlap with questions 4-5. | |  |  |  |
| **Findings:**  (-) Two patients found *'accomplishments’* awkward to describe on their own behalf.  (-) Three patients were not ‘*proud’* of anything and found the term ‘*strange*’ or ‘*overrated*’. One of the patients felt that the interviewer wanted her to be proud. | | 11 | 1.6 | 44%  (8/18) |
|  | | | | |
| ***Question 7: Are there particular things that you feel still need to be said to your loved ones,***  ***or things that you would want to take the time to say once again?*** | | | | |
| **Considerations:**  (+) Eight of the professionals had positive things to say about the questions relevance.  (-) Three professionals noted that the question had subtle hints to a limited time-horizon.  (-) Five professionals noticed an overlap with questions 3, 8, 9, 10. | |  |  |  |
| **Findings:**  (-) Two patients were occupied with the risk of hurting someone who was left out.  (-) One patient chose to give her answer in personal letters.  (-) Two patients refused to answer as soon as they heard “still need to be said”. | | 12 | 1.6 | 63%  (12/19) |
| ***Question 8: What are your hopes and dreams for your loved ones?*** | | | | |
| **Considerations:**  (+) Five professionals said that the formulation is good and praised the content in high tones.  (-) Three professionals said that the answer could overlap with the questions 3, 7, 9, 10 and 11. | |  |  |  |
| **Findings:**  (-) No comments from the patients.  (-) The therapists asked for hopes and dreams in relation to named persons 10 times. | | 13 | 2.0 | 90%  (24/26) |
|  | | | | |
| ***Question 9: What have you learned about life that you would want to pass along to others?*** | | | | |
| **Considerations:**  (+) Three professionals considered the question to be potent and extremely relevant for both patient and relatives.  (-) Two professionals noticed that the question demands a ‘grandiose’ answer and the focus on passing on life-lessons represents an ‘omniscient’ attitude.  (-) Two professionals noted some long formulations that could be shorter and clearer.  (-) One professional thought it overlapped with questions 3, 7, 8, 10 and 11. | |  |  |  |
| **Findings:**  (-) Three patients found it difficult and were overwhelmed either because it was unclear when they learned the most*,* or because they feared hurting or offending the receivers.  (-) One patient found it redundant. | | 12 | 1.4 | 81%  (13/16) |
|  | | | | |
| ***Question 10: What advice or words of guidance would you wish to pass along to your***  ***[son, daughter, husband, wife, parents, other(s)]?*** | | | | |
| **Considerations:**  (+) Three professionals considered the question to be ‘*fairly OK*’.  (-) Three professionals found the term “*words of guidance*’ artificial and technical.  (-) One professional found that implying the creation of something lasting beyond one’s own existence may be confronting.  (-) One professional thought there was an excessive amount of words in the question.  (-) Four professionals thought it overlapped with questions 3, 7, 8, 9 and 11. | |  |  |  |
| **Findings:**  (-) Two patients found it was inappropriate and felt incompetent telling others what to do.  (-) One patient found it redundant. | | 9 | 1.1 | 80%  (8/10) |
|  | | | | |
| ***Question 11: Are there words or even instructions you would like to offer your family,***  ***to help prepare them for the future?*** | | | | |
| **Considerations:**  (+)Three professionals found it ‘fairly ok’. One of them said it was better than question 10.  (-) One professional said that something lasting is implied, which may be confronting.  (-) Two professionals wondered how it is possible to ‘*prepare’* for a future in grief, and found the word inappropriate.  (-) The term '*instructions'* was criticised by two professionals for being too practical.  One professional said that ‘instructions’ can put stress on the relatives.  (-) Two professionals thought it overlapped with questions 3, 7, 8, 9 and 10. | |  |  |  |
| **Findings:**  (-) Five patients reacted to or were overwhelmed by ‘*messages and instructions*’ because they thought it would be a violation of the ‘free will’ of the receivers.  (-) One patient thought she had already answered this question. | | 6 | 1.3 | 63%  (5/8) |
|  | | | | |
| ***Question 12: In creating this permanent record, are there other things that you would like included?*** | | | | |
| **Considerations:**  (+) Three professionals found it to be a relevant and proper way of ending the interview.  (+) One professional thought that ‘*permanent*’ was a solemn word that underlined the value of the document.  (-) Two professionals thought that the word ‘*permanent*’ was too solemn and superfluous.  (-) One professional thought that the word *'document*' was associated with a legal text and therefore inappropriate.  (-) One professional thought that the phrase *'other things*' implied a change of subject that posed a risk of ending the interview abruptly. | |  |  |  |
| **Findings:**  No comments from patients. | | 12 | 1.3 | 44%  (7/16) |

* The table is based on data from (1) qualitative analysis of hypothetical interviews with professionals (N=10) (‘considerations’), (2) qualitative analysis of 20 DT transcripts (‘findings’), and (3) quantitative analysis of the same 20 DT transcripts. Predominantly positive statements are marked (+), and predominantly negative concerns are marked (-).

** Mean times asked per patient.

*** Total number of times answered divided by total number of times asked.
